# Specificity of plant-microbe interactions in the tree mycorrhizosphere biome and consequences for soil C cycling

**DOI:** 10.3389/fmicb.2014.00261

**Published:** 2014-06-03

**Authors:** Carolyn Churchland, Sue J. Grayston

**Affiliations:** Belowground Ecosystem Group, Department of Forest and Conservation Sciences, University of British ColumbiaVancouver, BC, Canada

**Keywords:** mycorrhizosphere, root exudates, plant-microbe interactions, LMWOA, signaling, carbon cycling, ectomycorrhizae, arbuscular mycorrhizae

## Abstract

Mycorrhizal associations are ubiquitous and form a substantial component of the microbial biomass in forest ecosystems and fluxes of C to these belowground organisms account for a substantial portion of carbon assimilated by forest vegetation. Climate change has been predicted to alter belowground plant-allocated C which may cause compositional shifts in soil microbial communities, and it has been hypothesized that this community change will influence C mitigation in forest ecosystems. Some 10,000 species of ectomycorrhizal fungi are currently recognized, some of which are host specific and will only associate with a single tree species, for example, *Suillus grevillei* with larch. Mycorrhizae are a strong sink for plant C, differences in mycorrhizal anatomy, particularly the presence and extent of emanating hyphae, can affect the amount of plant C allocated to these assemblages. Mycorrhizal morphology affects not only spatial distribution of C in forests, but also differences in the longevity of these diverse structures may have important consequences for C sequestration in soil. Mycorrhizal growth form has been used to group fungi into distinctive functional groups that vary qualitatively and spatially in their foraging and nutrient acquiring potential. Through new genomic techniques we are beginning to understand the mechanisms involved in the specificity and selection of ectomycorrhizal associations though much less is known about arbuscular mycorrhizal associations. In this review we examine evidence for tree species- mycorrhizal specificity, and the mechanisms involved (e.g., signal compounds). We also explore what is known about the effects of these associations and interactions with other soil organisms on the quality and quantity of C flow into the mycorrhizosphere (the area under the influence of mycorrhizal root tips), including spatial and seasonal variations. The enormity of the mycorrhizosphere biome in forests and its potential to sequester substantial C belowground highlights the vital importance of increasing our knowledge of the dynamics of the different mycorrhizal functional groups in diverse forests.

## Introduction

Soil organic matter (SOM) is the largest carbon (C) pool in terrestrial ecosystems (Falkowski et al., 2000; Fontaine et al., 2003), greater than terrestrial biomass C and atmospheric C combined (Jobbágy and Jackson, 2000). Carbon enters the SOM pool via litter (leaves, coarse and fine roots), brash (branches and coarse woody debris) and root exudates. The proportion of recently photosynthesized C allocated to leaves, storage, metabolism and root exudates has important consequences for soil C storage and varies depending on the environment, plant type, age of the plant, microbial symbionts and nutrient availability (Litton et al., 2007; Epron et al., 2012). Belowground C allocation is notoriously difficult to measure and varies depending on the spatial heterogeneity of belowground structures, the assemblage of microorganisms in the rhizosphere and environmental conditions (Subke et al., 2009; Kuzyakov and Gavrichkova, 2010; Mencuccini and Holtta, 2010; Warren et al., 2012). Recent studies have challenged our understanding of the mechanisms of C sequestration in soil. Clemmensen et al. (2013) showed that 50–70% of C stored in soil is derived from roots or root-associated microorganisms and that humus accumulation in boreal forests is regulated mainly by C allocation to roots and associated mycelium rather than decomposition of litter by saprophytes. Consequently, studies are beginning to focus on quantifying not only C allocation belowground, but also the spatial and temporal distribution of this C and how it is influenced by root-associated mycorrhizae (Litton and Giardina, 2008; Chapin et al., 2009; Warren et al., 2012).

Ninety percent of vascular plants form symbiotic relationships with mycorrhizal fungi (Wang and Qui, 2006; Smith and Read, 2008). Mycorrhizae can be generalized into two groups, endomycorrhizae, where hyphae penetrate root cells, and ectomycorrhizae, which do not penetrate. There are several types of endomycorrhizae including ericoid, arbutoid, monotropoid, orchid and, by far the most prevalent, arbuscular (occurring in approximately 85% of plant species) (Smith and Read, 2008). Arbuscular mycorrhizae (AM) are generally Glomeromycota, and form vesicles or arbuscules after invaginating the cell membranes of root cells. Ectomycorrhizae (ECM) are typically Basidiomycetes, Ascomycetes and Zygomycetes, occuring in 10% of plant species (mostly trees and woody plants). Ectomycorrhizae create a hyphal mantle covering the root tip and form a Hartig net within the root cortex, surrounding the root cells. Although saprotrophic fungi and bacteria are the primary decomposers in the soil, plant acquisition of released nutrients, such as N and P, is achieved through their symbiotic relationships with mycorrhizae (Read and Perez-Moreno, 2003; Lindahl et al., 2007; Talbot et al., 2008).

Mycorrhizae are involved in a number of important soil processes including: weathering of mineral nutrients (Landeweert et al., 2001; Finlay and Rosling, 2006; Wallander, 2006), C cycling, mediating plant responses to stress (Finlay, 2008), and interacting with soil bacteria (both negatively e.g., pathogens and positively e.g., mycorrhization helper bacteria) (Johansson et al., 2004; Frey-Klett et al., 2007). Ectomycorrhizae have broad enzymatic capabilities; they can decompose labile and recalcitrant SOM, and some can mineralize organic N (Chalot and Brun, 1998). This allows the mycorrhizae to transfer large amounts of N directly to their host plants (Hobbie and Hobbie, 2006). Arbuscular mycorrhizal fungal enzymatic capabilities are not thought to be as extensive as ECM; AM can only transfer small amounts of N to their hosts when soil-N levels are high (Tobar et al., 1994; Hodge et al., 2000; Govindarajulu et al., 2005; Reynolds et al., 2005). Arbuscular mycorrhizae mainly access inorganic N sources (Fellbaum et al., 2012), though organic N uptake by AM has been demonstrated in boreal forests (Whiteside et al., 2012). However, AM can transfer large amounts of P to their plant hosts (Smith and Read, 2008), either by hydrolysation of organic P from hyphal tips and subsequent transfer to the tree via arbuscules, or by uptake, conversion and transport of inorganic phosphorus along hyphae. Although some plant species can form symbiotic relationships with both AM and ECM, the dominance or presence of one over the other will alter tree-nutrient availability.

**Figure 1:**
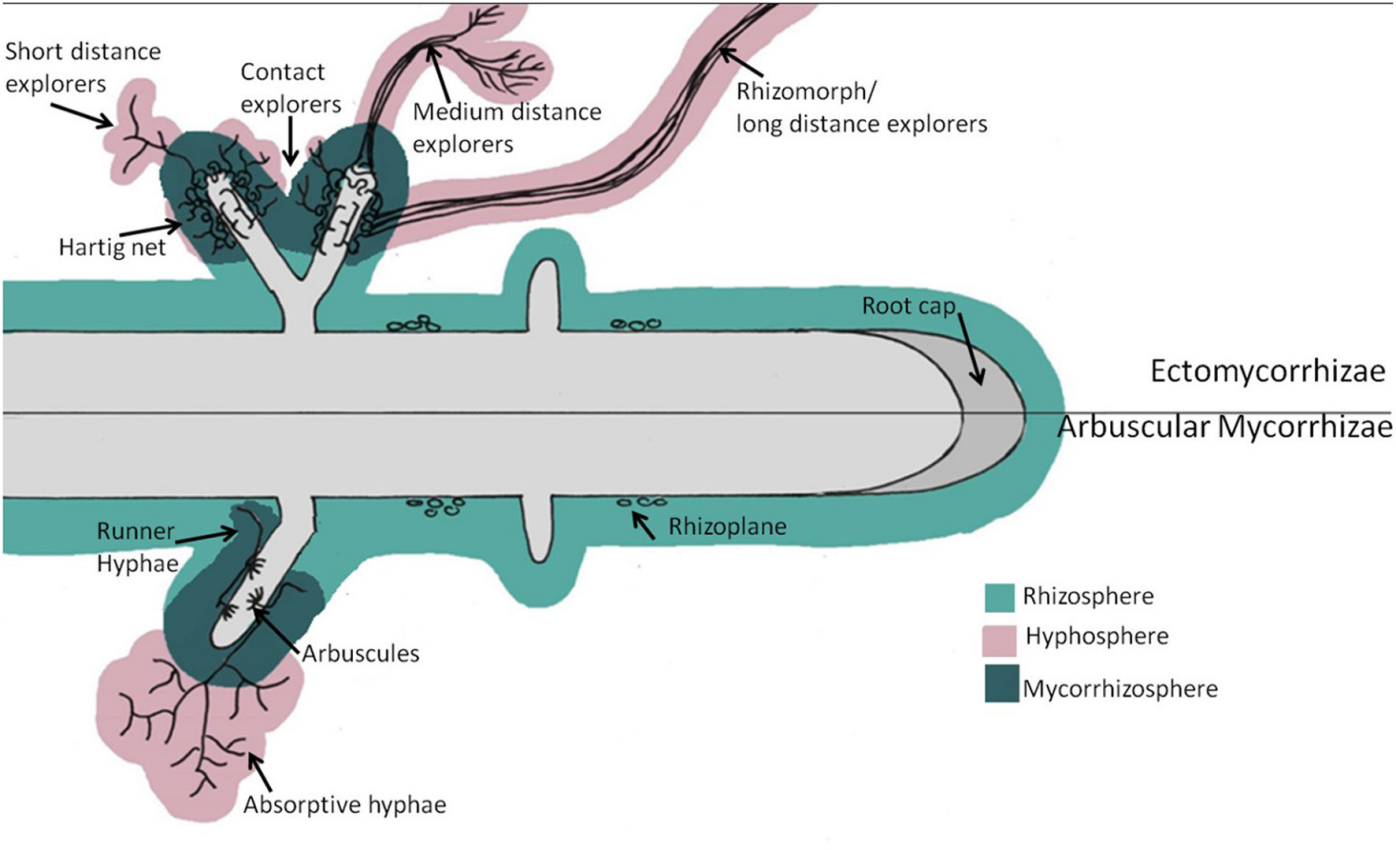
**Schematic view of root-mycorrhizal zones of influence and the various mycorrhizal growth forms**. Rhizoplane describes the area adjacent to the root where the soil particles adhere. The Rhizosphere is the area of soil around the root that is influenced by root-exuded labile C. The hyphosphere is the area of soil around mycorrhizal hyphae that is influenced by hyphal-exuded labile carbon and enzyme production. The mycorrhizosphere is the area of soil influenced by root and mycorrhizal communities combined.

There are 10,000 ECM fungal species that are known to be associated with as many as 8,000 different plant species (Taylor and Alexander, 2005). Tree species select mycorrhizae and free-living microorganisms through exudation of distinct chemical signals into the rhizosphere (the area surrounding the root that is directly influenced by root exudates, Figure 1) (Pires et al., 2012; Shi et al., 2012). Specific exudates will trigger the expression of mycorrhization genes, which are associated with the initiation of hyphal growth toward the plant root rhizosphere (Martin et al., 2007; Podila et al., 2009). In addition, there is increasing evidence that tree-species-rhizosphere community differences are the result of the trees “selecting” for specific microbes through root exudates (Prescott and Grayston, 2013). Plants release several types of root exudates including: mucilage that maintains a constant moisture environment, metal chelators that mobilize iron and zinc, and various forms of C comprising of carbohydrates, amino acids, low-molecular-weight aliphatic- and aromatic-acids, fatty acids, enzymes and hormones (Grayston et al., 1997; Table 1). The composition and quantity of root exudates will vary depending on tree species (Tuason and Arocena, 2009), and will also be modified within a given tree species depending on which mycorrhizal species colonize the tree roots (van Hees et al., 2005). Different ECM can increase root exudation of organic acid (van Hees et al., 2003, 2005; Johansson et al., 2009) and can change organic acid composition compared to non-mycorrhizal trees (Klugh and Cumming, 2003; van Hees et al., 2005). The variation in C allocated to ECM- and AM-roots, and subsequently ECM and AM root exudates is due, in part, to hyphal exudation from the mycorrhizae and mycorrhizal morphology. These hyphal exudates create an area of greater microbial biomass and activity, termed the mycorrhizosphere (area surrounding the mycorrhizal root tip) or hyphosphere (Figure 1) (Jones et al., 2004; Frey-Klett et al., 2007; Finlay, 2008; Nazir et al., 2010). Although bacteria and archaea are omnipresent in the rhizosphere and mycorrhizosphere, their role in ecosystem processes is only beginning to be understood.

**Table 1:** **Organic compounds and enzymes found in root exudates (Dakora and Phillips, 2002; Rasmann and Agrawal, 2008)**.

**Amino acids**	**Organic acids**	**Fatty acids**	**Sugar**	**Sterols**	**Growth factors and Vitamins**	**Purines. Nucleosides**	**Enzymes**	**Inorganic Ions and Gases**	**Phytochemical compounds**
α-alanine	Acetic	Linoleic	Arabinose	Compesterol	Biotin thiamine	Adenine	Acid/alkaline	HCO_3_^−^	Aldehyde
β-alanine	Aconitic	Linolenic	Deoxyribose	Cholesterol	Choline	Cytidine	Amylase	OH^−^	Alkaloid
γ-aminobutyruc	Aldonic	Oleic	Fructose	Sitosterol	Niacin	Guainin	Invertase	H^+^	Cardenolide
acid	Ascorcic	Palmitic	Galactose	Stigmasterol	Panthothenic	Uridine	Peroxidase	CO_2_	Cyanic
α-aminoadipic Acid	Benzoic	Stearic	Glucose		Pantothenate		Photophatase	H_2_	Glucoside
Arginine	Butyric		Maltose		Pyridoxine		Phenolase		Furanocoumarin
Asparagine	Caffeic		Mannose		Riboflavin		Polygalacturonase		Glcosinolate
Aspartic	Citric		Mucilage		p-amino benzoic		Protease		Glycoalkaloid
Citrulline	Erythronic		Oligosacchirides		acid				Hydroxamic acid
Cystathionine	Ferulic		Raffinose		N-methyl nicotinic				Iridoid glycoside
Cysteine	Formic		Rhamnose		acid				Phytoecdysteroid
Cystinemugineic	Fumaric		Ribose						Pyrrolizdine
Deoxymugineic	Glutaric		Sucrose						alakoid
3-epihydroxy	Glycolic		Xylose						Polyphenol
Glutamate	Glyoxilic								Resin
Glycine	Lactic								Tannin
Histidine	Malic								Terpenoid
Homoserine	Malonic								Triterpene
Isoleucine	Oxalic								
Leucine	Piscidic								
Lysine	Propionic								
Methionine	Pyruvic								
Mugineic	Succinic								
Ornithine	Syringic								
Pheylalalnine	Tartaric								
Praline	Tetronic								
Proline	Valeric								
Serine	Vanillic								
Theronine	p-coumaric								
Tryptophan	Oxalacetic								
Tyrosine	p-								
Valine	hydroxybenzoic								

This review focuses on describing host-specificity of soil microorganisms and fauna in the mycorrhizosphere of trees, the signals involved in establishing these interactions, and their impact on soil C flow and sequestration. We concentrate on interactions within the mycorrhizosphere, as this is the active site of root exudation, nutrient cycling, and plant nutrient uptake. The spatial enormity of the mycorrhizosphere biome in forests hints at its potential to sequester substantial amounts of C belowground. An understanding of the controls on C allocation belowground, and the movement of that C throughout the soil environment is a vital knowledge gap.

## The mycorrhizosphere biome

### Tree-mycorrhizal specificity

Many temperate forest tree species have ECM associations (including: pine, spruce, larch, hemlock, true firs, Douglas-fir, aspen, birch); some species have AM associations (e.g., cedar, maple, ash) and some have both (e.g., alder, poplar). Some tree species, such as Douglas fir (which associated with more than 2000 known ECM, Molina and Trappe, 1982) have high fungal receptivity, whereas other tree species such as alder (which only associate with 50 known ECM, Pritsch et al., 1997) have narrow fungal receptivity. It has been estimated that ECM mycelia can account for up to 80% of the fungal community and 30% of the total microbial biomass in forest soils (Högberg and Högberg, 2002; Wallander, 2006).

The presence and abundance of specific plant species can influence soil microbial community composition and function (Kourtev et al., 2002; Edwards and Zak, 2010; Eisenhauer et al., 2010), which can, in turn, impact soil C cycling and sequestration as mycorrhizal species differ in growth strategies and C demand. There is evidence of specificity in many plant-microbe interactions, suggesting both strong selective pressure and competition within the rhizosphere microbiome (Podila et al., 2009). There are many species of ECM fungi (Smith and Read, 2008) and though many ECM (e.g., *Lactarius*) have a broad host range some (e.g., *Suillus*) have only narrow host range (Bruns et al., 2002; Kennedy et al., 2003). The signaling specificity by host tree species to engage ECM fungi has been well studied (Molina and Trappe, 1982; Ishida et al., 2007; Tedersoo et al., 2008). For example, distinct chemical signals (e.g., small-secreted proteins and hydrophobins) may enable trees such as *Populus* to recruit advantageous ectomycorrhizal fungi from the broad soil microbial community (Podila et al., 2009). Whole-genome sequencing is now enabling us to have a much greater understanding of the suite of important genes and signals involved in ECM symbiotic associations (Martin et al., 2008, 2010). However, we are only just beginning to understand the factors involved in specificity and selection in AM associations (Brachmann and Parniske, 2006; Bonfante and Genre, 2010).

### Mycorrhizal morphology

Variations in extrametrical mycelium (EMM) hyphal pattern production and in mycorrhizae type may have consequences for C flow and carbon sequestration. ECM fungal taxa vary in the growth patterns of their EMM as a result of their multifarious foraging strategies (Agerer, 2001); the dominance of one morphological type over the other may have consequences for the spatial distribution of recent photosynthates belowground. Agerer (2001) describes the following ECM anatomies: contact explorers, convoy explorers, long-distance explorers, medium distance explorers and short-distance explorers (Figure 1). Contact explorers are EMM with a smooth mantle and few emanating hyphae (diffuse hyphal cords), the tips of which are often in close contact with dead leaves. Examples of contact explorers are *Lactarius* and *Russula* species that produce exudates throughout their hyphae. Convoy explorers are EMM that grow within rhizomorphs (aggregated parallel hyphal cords that can conduct nutrients over long distances) or mantles and produce haustoria in cortical cells of roots. Long-distance exploring EMM are smooth with highly differentiated rhizomorphs. For example, Boletales species are long distance hydrophilic hyphal explorers, and only exude compounds from their tips. Medium distance explorers have some rhizomorph formation and form 3 subtypes: fringe, mat, and smooth. Fringe subtype hyphae fan out from hairy rhizomorphs, which ramify and interconnect (e.g., *Dermocybe cinnamomeolutea*). Mat subtype hyphae have a limited range of exploration and rhizomorphs do not differentiate (e.g., *Hysterangium stoloniferum*). Smooth subtype hyphae have internally undifferentiated rhizomorphs with a central core of thick hyphae, with smooth mantles, and a few emanating hyphae (e.g., *Thelephora terrestris*) (Agerer, 2001). Short-distance explorers have a voluminous envelope of emanating hyphae without rhizomorph formation (e.g., *Quercirhiza squamosal*) (Agerer, 2001).

**Figure 2:**
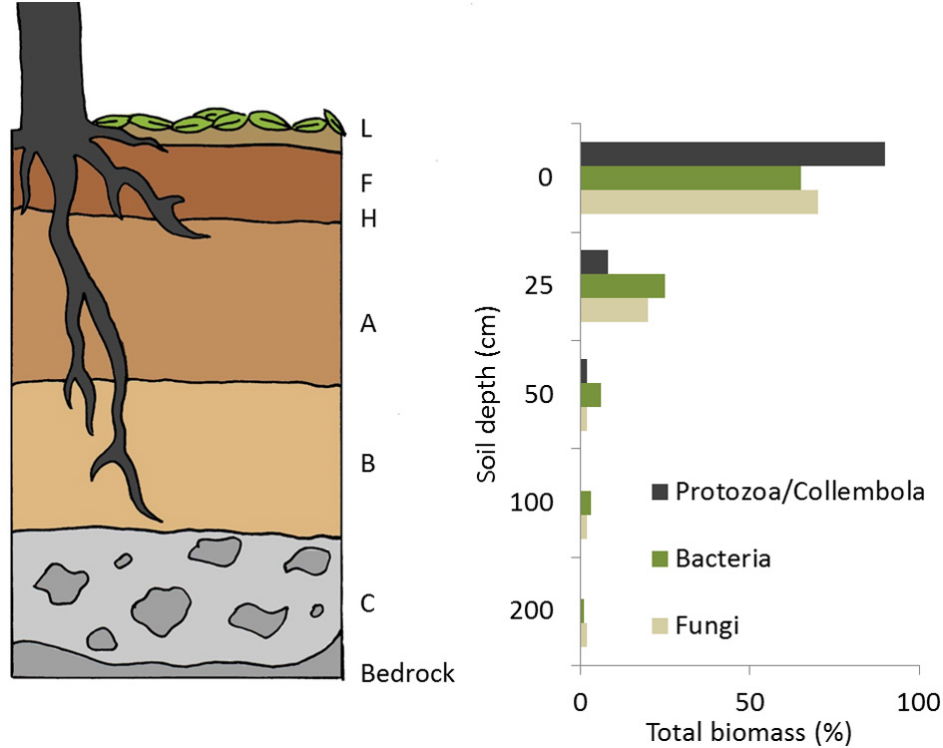
**Diagram of soil profiles with depth and the relative proportion of collembola/protozoa, bacteria and fungi at each of these depths**. L, characterized by the accumulation of organic matter; F: characterized by the accumulation of partially decomposed organic matter; H: characterized by the accumulation of decomposed organic matter where the original structure is indescernable. A: mineral horizon characterized by eluviation of materials in solution, or accumulation of organic matter, or both. B: mineral horizon characterized by enrichment of clay, organic matter, and iron and aluminium oxides or by *in situ* weathering. C: mineral horizon characterized by little or no alteration through the soil-forming processes, usually represents the parent material.

Hyphae have the ability to move carbon both horizontally, over long distances, extending well beyond the roots of trees and vertically, down the soil profile. Most ECM are found in the F and H soil layer (area of highly decomposed leaves beneath surface of forest floor, Figure 2), but also can be found in the mineral soil, whereas other ECM prefer decaying wood (Amaranthus and Perry, 1989; Tedersoo et al., 2003). Some ECM are able to mobilize minerals from rocks in soil (Landeweert et al., 2001), whereas others access nutrients from coarse woody debris (Amaranthus et al., 1994). Some ECM fungi also have saprophytic growth capabilities e.g., *Tomentella* sp. (Kõljalg et al., 2000). It is hypothesized that these ECM may switch to a saprophytic lifestyle when photosynthate C becomes scarce e.g., during winter (Courty et al., 2008).

Arbuscular mycorrhizae do not form rhizomorphs and are considered to have five distinct hyphal architecture types (Figure 1). These include: infection networks, produced by spores and root fragments; germ tubes (only 20–30 mm long); hyphal bridges that connect runner-type hyphae and form patches of dense hyphal networks close to the root zone (Friese and Allen, 1991; Dodd et al., 2000); runner-types that expand rapidly through the soil or along roots (Mosse, 1962), seeking out new segments of roots to infect (Friese and Allen, 1991); and absorptive hyphal networks that explore the soil matrix for nutrients (Friese and Allen, 1991). Absorptive hyphal networks can extend 4–7 centimeters into the soil. Each network can have up to 8 branching orders, with each branch extending approximately 5 millimeters (Allen, 2007). Bago et al. (1998) described a 6th architectural form, where absorptive hyphae can form from runner hyphae, extending the potential range of nutrient absorption well beyond 4–7 centimeters. However, ECM EMM can extend even further from the roots as a result of rhizomorph formation. ECM rhizomorphs live, on average, 11 months, but have been observed to live for up to 7 years (Treseder et al., 2005). In contrast, AM hyphae only live on average 5–6 days (Staddon et al., 2003), suggesting the ECM dominated forests have greater C storage potential. The following section describes tree-rhizosphere C flow in greater detail.

## Quantification and characteristics of mycorrhizosphere C flow

### Tree-rhizosphere C flow

Differences in root-associated fungi (both the presence/absence and type of fungal association) may be responsible for the large variation (10 X) in root exudation rates (Phillips et al., 2008, 2011). Exudation rates from root tips and hyphal tips tend to be greatest in the fine roots and in mycorrhizae that are allocated more C (Phillips et al., 2008, 2011). Carbon allocation to ECM hyphae will vary depending on ECM taxa (Bidartondo et al., 2001) and stage of colonization. For example, more C is allocated belowground during early stages of colonization (Cairney et al., 1989; Cairney and Alexander, 1992). Movement of recent photosynthates within EMM is not uniform, and will vary depending on the fungal species and their life stage (Cairney, 2012). Sun et al. (1999) demonstrated that ECM hyphal tips were active sites of exudation and re-adsorption of compounds, with little exudation along rhizomorphs. In addition, Leake et al. (2001) showed that more C was allocated to frontal tips of hyphae that occupied a hotspot of organic matter in soil. Infected ECM root tips may receive 42 times more carbon than uninfected root tips on the same plant (Cairney et al., 1989; Wu et al., 2002). Therefore there will be much patchiness in root exudate distribution in the forest floor, depending on root distribution and hyphal distribution.

Several techniques- including tree-girdling and stable-isotope labeling- have the potential to accurately measure the amount of C allocated belowground as well as the impact of root-exuded C on the microbial community. Tree girdling has demonstrated that labile C drives soil respiration (Högberg et al., 2001). Tree girdling stops the flow of photosynthates to tree roots, altering the availability and quality of C sources available to soil microbes in the rhizosphere (Subke et al., 2004; Högberg et al., 2007). However, how girdling affects the soil microbial community, particularly the bacterial community, is not consistent. Tree girdling caused significant decreases in the activity and biomass of the soil microbial community in boreal and temperate forests (Scott-Denton et al., 2006; Weintraub et al., 2007); this was mainly due to loss of ECM (45% decrease in ECM biomass relative to non-girdled plots) (Högberg and Högberg, 2002; Yarwood et al., 2009; Pena et al., 2010). The response of bacterial abundance and biomass to girdling has been marginal in boreal forests (Högberg et al., 2007; Yarwood et al., 2009) and in sub-tropical evergreen broadleaf forests (Li et al., 2009). Koranada et al. (2011) observed (using PLFA) a significant reduction in fungal biomass and Gram-positive bacterial biomass in girdled beech forests. As Gram-positive bacteria were less affected by exudates, Koranada et al. (2011) hypothesized that other effects of girdling treatments on rhizospheric conditions, such as alterations in oxygen supply, pH and redox potential (a result of the reduced root respiration or uptake of nutrients by plants) may have decreased Gram-positive bacterial populations. Other studies have shown no effect of girdling on microbial biomass or soil respiration; however in some of these studies trees re-sprouted (e.g., Eucalyptus), (Wu et al., 2011; Chen et al., 2012), and in other studies carbohydrates were still available in roots after girdling (Binkley et al., 2006). The increased availability of root carbohydrates may lead to a positive priming effects on SOM decomposition, increasing microbial community biomass and activity in the short-term (Subke et al., 2004; Scott-Denton et al., 2006). The variability of tree-girdling results may be the result of variation in tree-mycorrhizal species associations, or may be due to priming effects. Consequently developing non-destructive techniques may provide more insight into C-flow in forest ecosystems.

Natural-abundance stable-isotope ratios have recently been used to non-destructively investigate the flux of C from trees to the soil microbial community. In a second-growth coastal western hemlock forests in B.C. eighty-year-old Douglas-fir and western hemlock trees supplied C to the mycorrhizal symbionts for a distance up to ten meters (Churchland et al., 2013). Similarly, labeling of young trees with ^13^C-enriched CO_2_ has also been used to assess spatial and temporal C flux belowground. Epron et al. (2011) showed that there was rapid transfer of recent photosynthates to the mycorrhizosphere of beech (0.5–1 day), oak (0.5–1 day) and pine (1–2 days), and that the patterns of carbon allocation belowground varied seasonally in pine and beech, according to the phenology of the species. Similarly, Esperschütz et al. (2009) demonstrated using ^13^CO_2_ pulse-labeling and PLFA analysis, that the C in beech root exudates is first utilized by Gram-negative bacteria and mycorrhizal fungi. Stem-injection-labelling of mature trees has shown that C exudation from 22-year-old Sitka spruce in the field is rapid (24 h) and that these exudates are utilized first by fungi. The extent of influence of these trees exudates can be up to 20 m away from the base, and may, in part, be due to transport through EMM (Churchland et al., 2012). Although these techniques are too coarse to measure carbon movement in a single hypha, they show that C can move great distances away from the tree base and are utilized by the fungi and bacteria in the rhizosphere.

### Root exudates

Characterizing root exudation is challenging, but new techniques hold potential for breakthroughs. Most studies characterizing exudates released by different tree species have been microcosm studies conducted on seedlings in the laboratory under controlled conditions, either in hydroponic or sand systems, which do not scale up to mature trees and forests (Grayston et al., 1997). Hydroponic systems lack the physical substrates important for root growth; this affects exudation and can lead to re-uptake of exudates by plant roots. Studies in sand or soil systems are limited because of adsorption of exudates or degradation by the microbial community (Grayston et al., 1997, and references therein). There have been a few studies of tree root exudation in the field, mainly on young seedlings using either excavated root tips (which are surface-sterilized and placed in sterile tubes in the field) or soil extraction techniques. This latter approach has similar problems to the microcosms mentioned above (Phillips et al., 2008). In addition, it is difficult to extrapolate exudation rates from seedlings to mature trees, as a smaller portion (though, in total, a much greater amount) of recently-photosynthesized C is being allocated to the roots. Recently Shi et al. (2012) demonstrated an anion exchange membrane system that improved root exudate collection *in situ* from two-year-old radiata pine trees growing in large-scale biotrons. Because these anion exchange membranes rapidly adsorb root exudates there is little chance for consumption by microbes present in the biotron soil. This technique may result in a better understanding of root exudation from mature trees and forest stands.

The amount of C allocated to roots, root exudates, mycorrhizae and other rhizosphere microorganisms can change under different nutrient regimes and increase in the presence of specific microorganisms (Grayston et al., 1997). Ectomycorrhizal fungi influence both the quantity of C allocated to their roots, and the chemical composition of those exudates (van Schöll et al., 2006; Rineau and Garbaye, 2010). For example, ECM trees will allocate a third more C to their roots than non ECM trees (Durall et al., 1994; Rygiewicz and Anderson, 1994; Qu et al., 2004), likely because EMM have a large C demand (Rygiewicz and Anderson, 1994; Cairney and Burke, 1996; Cairney, 2012). Laboratory studies have shown that up to 29% of plant-assimilated C can be allocated to EMM (Rygiewicz and Anderson, 1994; Ek, 1997; Bidartondo et al., 2001). Environmental conditions also influence the degree to which tree roots are colonized, and likely mediate fluxes of labile C in forest soils (Meier et al., 2013). For instance, loblolly pine mass-specific exudation rates can vary by over three orders of magnitude under varying CO_2_ concentrations (Phillips et al., 2008, 2011). Plants have been observed to allocate more C to their roots and mycorrhizal symbionts under nutrient poor conditions (Zak et al., 1993; Franklin et al., 2012). In systems that are not N-limited, or in systems where N has been added, fungal biomass can decrease up to 45%, mainly due to decreased C allocation from trees to the mycorrhizal fungi (Högberg et al., 2007).

Root exudates represent semi-continuous input of labile C into soil, though exudation rates vary in time and space (Hinsinger et al., 2005), between deciduous and conifer species, over seasons (Collignon et al., 2011) and in different climates (Lin et al., 1999; Jones et al., 2004). Reviews on rhizodeposition from plants acknowledge the scant information on the character of exudates from trees (Grayston et al., 1997; Kuzyakov and Domanski, 2000; Neumann and Romheld, 2001; Jones et al., 2004). Plants are able to influence not only the quantity but also the composition of C exuded by their roots. This is thought to play a role in tree-microbe signaling and specificity in the rhizosphere. Production of enzymes, low-molecular-weight organic-acids (LMWOA), and other compounds support rhizosphere microbial communities (Bais et al., 2006). Root exudates also enhance nutrient availability by mobilizing poorly-soluble mineral-nutrients (Jones and Darrah, 1994; Marschner et al., 2011) and supplying labile-C substrates that increase rhizosphere microorganism activity and turnover (Phillips et al., 2012), ultimately influencing the decomposition of SOM (Rosling et al., 2004a,b). Most of the knowledge about the character of root exudates and how they may vary between tree species is on LMWOA and with ECM fungi (Cairney, 2012). In one of the few studies on carbohydrate characterization, Liebeke et al. (2009) used a gas-chromatograph-mass-spectrometer to reveal differences in the sugar content of soil extracts from different forest soils, demonstrating that oak soil contained mannitol and trehalose that was not present in beech soil. They hypothesized that the variation in sugar concentrations was responsible for differences in the bacterial communities under these tree species. There is increasing evidence that trees can actively restrict carbohydrate flow to their fungal partners. This is done through control of sucrose export and hydrolysis if the fungal partner does not deliver sufficient mineral nutrients (see review by Nehls et al., 2010).

### Plant-mycorrhizae signaling molecules

Several root exudates and hyphal exudates have the potential to induce mycorrhizal infection and change the microbial community structure of the rhizosphere. Secreted proteins, specifically a class of secreted proteins called effectors, have recently been established as plant-mycorrhizal signaling molecules (Lowe and Howlett, 2012). Effector proteins facilitate infection by suppressing immunity and/or inducing defense responses in plants (DeWit et al., 2009). For example, *Laccaria bicolor* was found to secrete the effector Mycorrhizal-Induced Small Secreted Protein 7 (MISSP7) during root colonization, in response to diffusible signals exuded from plant roots (Plett et al., 2011). Secretion and uptake of MISSP7 by the plant (via PI-3-P mediated endocytosis) affected cell wall chemistry, ultimately allowing hyphal penetration of the root apoplast. MISSP7 is the most upregulated protein during mycorrhization, and without it symbiosis does not occur (Plett et al., 2011). Following this discovery another effector protein, SP7, was uncovered (Maffei et al., 2012). Secreted by the AM fungi *Gigaspora intraradices*, SP7 interacts with a plant pathogenesis related transcription factor. SP7 was found to play a role in managing the formation of symbiosis with plant roots through the suppression of the plant immune system (Kloppholz et al., 2011). Plants have also been found to increase production of strigolactones under nutrient poor conditions (Maffei et al., 2012). Strigolactones have been found to induce fungal spore germination (Maffei et al., 2012) and hyphal branching (Bonfante and Requena, 2011), suggesting that plants might be signaling nearby mycorrhizae to promote infection. Much less is known about AM signaling, although recently it has been shown that AM fungi also produce active diffusible signals, similar to Nod factors released by rhizobia. These signals are needed for mycorrhizal formation (Bonfante and Requena, 2011). Similarly plant secreted effectors have also been found, which influence interactions between plant roots and free-living microorganisms (Hogenhout et al., 2009).

### Modifications by ECM/AM on exudates and signals

Mycorrhizae modify the amount and composition of root exudates (van Schöll et al., 2006; Johansson et al., 2008, 2009), affecting exudation into the mycorrhizosphere and hyphosphere (Sun et al., 1999; Ahonen-Jonnarth et al., 2000; Jones et al., 2004; Johansson et al., 2008, 2009). The tips of growing ECM hyphae have been found to exude sugars, polyols, amino acids, peptides, proteins, hydroxamate siderophores, various LMWOA and pigments (growing front; Table 1) (Sun et al., 1999; Ahonen-Jonnarth et al., 2000; Jones et al., 2004; Johansson et al., 2008, 2009). Different ECM taxa vary the amount and composition of compounds exuded (Lapeyrie et al., 1987; Griffiths et al., 1994; van Schöll et al., 2006; Johansson et al., 2009; Tuason and Arocena, 2009). In general, the presence of ECM increases organic acid exudation (Johansson et al., 2008, 2009) and/or changes the type of organic acid exuded (van Schöll et al., 2006; Table 2). For example, van Hees et al. (2006a) found that *Hebeloma crustuliniforme* (ECM), when in symbiosis with *Pinus sylvestris*, exuded oxalate and ferricrocin and, to a lesser extent, malonate and acetate which were absent from non-mycorrhizal Scots pine soil.

**Table 2:** **Modification of low molecular weight organic acid (LMWOA) exudates from trees by different ectomycorrhizal (ECM) and arbuscular mycorrhizal (AM) fungi**.

**Tree species**	**ECM or AM**	**Mycorrhizal symbiont**	**ECM/AM effect on LMWOA exudation (vs. non ECM/AM roots)**	**Methodology**	**References**
Scots pine	ECM	*Paxillus involutus*	↑ oxalic acid, formic acid	9-month-old inoculated seedlings were planted in sterilized soil collected from an E-horizon, and placed in climate controlled growth room. LMWOA were collected via suction from soil column and indentified using capillary zone electrophoresis	van Hees et al., 2005
		*Suillus granulatus*	↑ citric acid		
Norway spruce	ECM	*Paxillus involutus*	↑ malonic acid		
Scots pine	ECM	*Suillus variegatus*	↑oxalic acid	9–12-week-old inoculated seedlings were grown in petri dishes containing glass beads with a growth solution. LMWOA were analyzed using HPLC analysis	Ahonen-Jonnarth et al., 2000
		*Rhizopogon roseolus*	↑ oxalic acid		
		*Paxillus involutus*	↑oxalic acid, malonic acid		
Scots Pine (under elevated CO_2_)	ECM	*Sullius variegates*	↑oxalic acid	16-week-old inoculated seedlings were grown in petri dishes containing peat:vermiculite substrate with a growth solution. LMWOA were analyzed using HPLC analysis	Johansson et al., 2009
		*Sulliusbovinus*			
		*Paxillusinvolutus*			
		*Rhizopogon roseolus*			
		*Hebelomavelutipes*	↑citric, fumaric, formic, malonic acid		
		Piloderma croceum			
Scots pine	ECM	*Hebeloma longicaudum*	↓malonic acid	21-week-old inoculated seedlings were grown on glass beads or sand with a growth solution. LMWOA were indentified using capillary zone electrophoresus	van Schöll et al., 2006
		*Paxillus involutus*	↑oxalic		
		*Piloderma croceum*			
White spruce	ECM	*Not identified*	↑ malonic, oxalic, gluconic, succinic, protocatechuic acid	Soil collected *in situ* around trees that were 20–35 cm diameter at breast height. LMWOA were indentified using capillary zone electrophoresis	Tuason and Arocena, 2009
Subalpine fir	ECM	*Not identified*	↑ malonic, oxalic, glutaric, isocitric acid		
Norway spruce	ECM	*Paxillus involutus*	↑ Malate, citric	9-month-old inoculated seedlings were planted in a soil-sand column system. LMWOA were collected using suction from soil column and analyzed using capillary zone electrophoresus	van Hees et al., 2003
Scots pine	ECM	*Hebeloma crustuliniforme*	↑oxalic, citric, propionic acid	16-week-old inoculated seedlings were grown in a sand culture system. LMWOA were collected via suction from soil column and analyzed using capillary zone electrophoresis.	van Hees et al., 2006a
			↑oxalic acid, ferrocrocin	16-week-old inoculated seedlings were grown in aseptic multi-compartment dishes containing sterile nutrient agar with stock nutrient solution. LMWOA were analyzed using capillary zone electrophoresis	van Hees et al., 2006b
Norway spruce	ECM	*Laccaria bicolor*	↑oxalic acid	8-week-old inoculated seedlings were grown in glass bead mesocosms with growth medium. LMWOA were analyzed using HPLC analysis	Eldhuset et al., 2007
Japanese red pine	ECM	*Pisolithus tinctorius*	↑citric acid	4-month-old inoculated seedlings were grown in perlite in pots. LMWOA were analyzed using an electroconductivity detection method	Tahara et al., 2005
Scots pine	ECM	*Amantia muscaria*	↑LMWOA exudation, individual OA’s varied depending on N addition and elevated CO_2_	4-week-old Inoculated seedlings were grown in petri dishes containing vermiculite and a growth medium. LMWOA were analyzed using capillary zone electrophoresis	Fransson and Johansson, 2010.
		*Hebeloma velutipes*			
		*Piloderma fallax*			
		*Suillus variegatus*			
Scots pine	ECM	*Hebeloma velutipes,*	↑LMWOA exudation, especially oxalic acid	16-week-old inoculated seedlings were grown in petri dishes containing peat:vermiculate and growth medium. LMWOA were analyzed using capillary zone electrophoresis	Johansson et al., 2008
		*P. involutus,*			
		*Piloderma byssinum,*			
		*R. roseolus,*			
		*S. bovinus*			
		*S. variegatus*			
Tulip poplar	AM	*Acaulospora*	-	5-month-old seedlings were grown in fungal inoculated sand. Roots were washed for organic acid profiles. LMWOA were indentified using ion chromatography	Klugh and Cumming, 2007
		*morrowiae*	-		
		*Glomus*	↑ malate, citric acid		
		*claroideumG. clarum*	-		
		*Paraglomus brasilianum*	-		

↑, *increase;* -, *no change;* ↓, *decrease; n/a, information not available*.

There is some evidence that hyphal exudates result in specific hyphosphere bacteria communities (Table 3; See Nazir et al., 2010 for list of bacterial-AM fungal relationships). It has been suggested that organic acids contribute to microbial selection in the mycorrhizosphere (de Boer et al., 2005). Differences in LMWOA ECM hyphal exudation are thought to be partially responsible for selecting specific microbial communities (Martin et al., 2008; Tuason and Arocena, 2009). Similarly, Toljander et al. (2007) found increased γ-proteobacteria abundance when extracted AM mycelial exudates were present, including formate, acetate, α and β glucose, and oligosaccharides. Trehalose has been reported to select specific bacterial communities in the mycorrhizosphere of several tree species including, Douglas-fir, Corsican pine and oak (Frey et al., 1997; Rangel-Castro et al., 2002; Izumi et al., 2006a,b; Uroz et al., 2007). Frey et al. (1997) suggested that the release of trehalose by the ECM fungus *Laccaria bicolor* exerts a nutrient-mediated selection on the surrounding bacteria. Specifically, trehalose has been found to have growth-promoting effects on the mycorrhization-helper bacteria (MHB), *Pseudomonas monteilii*, when inoculated with the ECM fungus *Pisolithus albus* in a plate-assay (Duponnois and Kisa, 2006). Trehalose released by the mycelium of *Laccaria bicolor* was shown to be a chemoattractant for *Pseudomonas fluorescens* BBc6R8 (Frey-Klett et al., 2007). At present it is not clear how hyphosphere microbial communities will impact a mycorrhizal's ability to acquire nutrients, but it is clear that exudation specificity has the potential to select for species-specific microbial communities.

**Table 3:** **Examples of mycorrhization helper bacteria, with significant effects on ECM formation.**

**Fungi**	**Bacteria**	**MHB effect**	**Host plant**	**References**
*Laccaria laccata*	*Agrobacterium radiobacter*	↑ mycorrhizal colonization	6-month-old pine and birch seedlings grown on sand-mica-rock substrate	Leyval and Berthelin, 1993
*Suillus grevillei*	*Pseudomonas fluorescens* strain 70	↑ Fungal growth	Fungi and bacteria were cultured from sporocarps found in Eurpoean larch forest	Varese et al., 1996
	*Pseudomonas putida* strain 42			
*Geopora* species	*Sphingomonas* sp. 23L	↑ fungal inoculation, and tree growth	Willow tree cuttings potted in 1 kg of fly ash, bacterial inoculant was added	Hrynkiewicz et al., 2009
*Lactarius rufus*,	*Paenibacillus* sp. EJP73,	Altered root branching	Scots pine seedlings grown in vermiculite-peat moss microcosms	Aspray et al., 2006
*Laccaria bicolor* or	*Burkholderia* sp. EJP67,	↑*L. bicolor* mycorrhiza formation		
*Suillus luteus*	*Paenibacillus* sp. EJP73			
*Suillus granulatus*	*Ralstonia basilensis*,	Increased hyphal growth	1-week-old Japanese black pine was planted in autoclaved soil before inoculated with fungi	Kataoka et al., 2009
*Cenococcum geophilum*	*Bacillus subtilis*			
*Laccaria bicolor* S238N	*Pseudomonas fluorescens* BBc6R8	Promotes presymbiotic fungal-survival and increases radial growth, hyphal apex density and branching angle	Pre-symbiotic, grown on Pachlewski medium	Deveau et al., 2007
Amantia muscaria	*Streptomycetes* nov. sp. 505	1.2–1.7 fold increase in second-order root mycorrhizal rate	4-weeks-old Norway spruce and Scots pine seedlings were grown on autoclaved peatmoss and perlite before inoculation	Schrey et al., 2005
Suillus bovinus	*Streptomyces annulatus* 1003 (AcH 1003)			
*Laccria laccata*	*Pseudomonas* species, *Bacillus* species	↑ mycorrhizal colonization	Douglas-fir seeds were sown in inoculated vermiculite-peat moss polythene cells	Duponnois and Garbaye, 1991
*Laccaria fraterna*	*Bacillus* species	↑ mycorrhizal colonization	Eucalyptus seeds were sown in sphagnum peat-perlite before inoculation	Dunstan et al., 1998
*Laccaria laccata*	*Pseudomonas* species			
*Lacterius rufus*	*Paenibacillus* species	↑ mycorrhizal colonization	Sterile Scots pine seedlings grown on agar petri dishes were used for inoculation once roots were 4.5–6 cm long	Poole et al., 2001
	*Burkholderia* species			
*Pisolithus alba*	*Pseudomonas monteilii*	↑ mycorrhizal colonization	Soapbush seedlings were planted in autoclaved soapbush soil before inoculation	Founoune et al., 2002a
	*Pseudomonas resinovorans*			
*Pisolithus* species	*Pseudomonas* species	↑ mycorrhizal colonization	Soapbush seedlings were planted in autoclaved soapbush soil before inoculation	Founoune et al., 2002b
*Rhizopogon luteolus*	Unidentified	↑ mycorrhizal colonization	Radiata pine seedlings were grown on autoclaved soil before inoculation	Garbaye and Bowen, 1989
*Scleroderma* species	*Pseudomonas monteilii* strain HR13	↑ mycorrhizal colonization	Acacia seedlings were grown on sterilized sand before inoculation	Duponnois and Plenchette, 2003
*Pisolithus* species				
*Suillus luteus*	*Bacillus* species	↑ root growth and mycorrhizal colonization	2-week-old Scots pine seedlings were grown on inoculated peat-vermiculate petri dishes	Bending et al., 2002

### Spatial and seasonal variation in rhizosphere C flow

Rhizosphere C flow varies spatially down the soil profile and horizontally with changes in root and hyphal distribution. Carbon flow also fluctuates seasonally and differs between coniferous and deciduous trees. In forest soil there is soil microbial community-composition stratification with depth due to a decrease in root biomass, root exudates, available C and a shift in SOM composition (Grayston et al., 1997; Berg et al., 1998; Fritze et al., 2000; Leckie et al., 2004; Lejon et al., 2005). Fungi are typically found in upper soil layers (Litter>Formulating >Humified, Figure 2) (Gardes and Bruns, 1993; Hirose et al., 2004). In contrast, actinomycete abundance has been shown to increase with depth (Fritze et al., 2000) while Gram–negative bacterial distribution is linked to root distribution (Soderberg et al., 2004). However, soil respiration rates and microbial activity are related to proximity to trees and tree roots (Churchland et al., 2013). In a free-air carbon-dioxide-enrichment (FACE) study, Phillips et al. (2008) showed that exudation rates could be predicted by the number of roots and mycorrhizal fine root tips (Pritchard et al., 2008b). This suggests that recent tree-carbon can be transported over large distances via roots and hyphae, supporting microbial communities meters away from the tree base.

There are different seasonal and physiological effects on rhizosphere C flow for deciduous and evergreen tree species. In a meta-analysis of C-allocation dynamics in trees Epron et al. (2012) showed that broadleaf trees exhibit, on average, 10 times higher rates of C transfer than coniferous species, although this varies depending on season. In spring (before bud break) and fall (during leaf senescence), broadleaves allocate a greater proportion of C to their roots (Epron et al., 2012). A number of studies have documented seasonal trends in soil microbial communities and activities in a variety of ecosystems (Allison and Treseder, 2008; Björk et al., 2008; Cruz-Martinez et al., 2009), including the coniferous forests of the Pacific Northwest (Brant et al., 2006; Moore-Kucera and Dick, 2008) and deciduous forests of Europe (Hibbard et al., 2005; Rasche et al., 2011). Studies specifically examining ECM fungi have found that their community structure, as well as enzymatic and metabolic capabilities, exhibit considerable temporal variation over a single year (Buée et al., 2005; Courty et al., 2007, 2008). This is likely related to differences in belowground C flow (Collignon et al., 2011). Burke et al. (2011) showed ECM, but not AM varied over a growing season in a mixed deciduous forest in Pennsylvania and that ECM and AM were associated with different enzyme activities involved in nutrient cycling. Specifically, AM fungi were associated with leucine aminopeptidase and urease, both enzymes involved in N acquisition. Arbuscular mycorrhizae were not traditionally considered able to supply their host with significant amounts of N (Smith and Read, 2008), though there is recent evidence that AM fungi can access both inorganic N (Fellbaum et al., 2012) and organic N (Whiteside et al., 2012) sources in forests. ECM were associated with most measured enzymes involved in C and N acquisition, but only during the late summer (Burke et al., 2011). This indicates that mycorrhizal ability to breakdown recalcitrant C and provide their host with N may vary seasonally.

## Effects of mycorrhizosphere C flow on other organisms

### Free-living fungi, bacteria and archaea

It is clear that variation in the quality and quantity of C released in root and hyphal exudates produced by different tree species can result in different rhizosphere and hyphosphere microbial communities and this varies between tree species associated with ECM and AM (Garbaye, 1991; Broeckling et al., 2008; Prescott and Grayston, 2013). Phillips and Fahey (2006) collected rhizosphere soil, bulk soil, and fine roots from the upper four centimeters of 12 monospecific tree species plots (six AM and six ECM tree species) planted on a common soil. The rhizosphere of AM trees and ECM trees were 10–12 and 25–30% more active (as measured by respired CO_2_) than bulk soil, demonstrating that ECM trees have a greater rhizosphere effect than AM trees. The magnitude of rhizosphere effects was negatively correlated with the degree of mycorrhizal colonization in AM tree species and with fine root biomass in ECM tree species. This suggests that different factors influence rhizosphere effects in tree species forming AM vs. ECM associations (Phillips and Fahey, 2006). Hyphal exudates from ECM tips support a diverse population of bacteria, archaea and fungi (Frey-Klett et al., 2007; Tedersoo et al., 2009; Bomberg et al., 2011). High throughput sequencing methods developed over recent years are enabling us to obtain much greater phylogenetic resolution to our studies of mycorrhizosphere microbial communities. For example, Kluber et al. (2010) used DNA sequencing to identify the rhizomorphic ECM mat-forming taxa (*Hysterangium, Piloderma, Suillus* and *Russula* species) in the forest floor and the hydrophobic mat-forming taxa (*Gomphus* and *Ramaria* species) in the mineral soil in a Douglas-fir forest. The two ECM mat forms had enhanced enzyme activities, specifically chitinase, phosphatase and phenol oxidase compared to non-mat forms in adjacent locations (Kluber et al., 2010). It was not established if the enhanced enzyme activity in the mats was the result of the ECM themselves or the distinctive bacteria and fungi in their mycorrhizosphere (Kluber et al., 2011). Bomberg and Timonen (2007, 2009) demonstrated (using PCR-DGGE of archaeal 16S rRNA genes) that there were specific archaeal communities in the ectomycorrhizosphere of several common boreal forest trees and that the type of ECM had the most influence on archaeal diversity. Bomberg et al. (2011) found no evidence of archaea in bulk humus samples lacking tree roots or ECM, indicating archaea are dependent on plant-derived C for growth. Similarly, Pires et al. (2012) used pyrosequencing and PCR-DGGE to reveal differences in archaeal richness between two mangrove species. Uroz et al. (2012) revealed (pyrosequencing 16S rRNA) that *Alpha*-, *Beta*-, and *Gammaproteobacteria* were significantly higher in the ectomycorrhizosphere of oak than in bulk soil and the bacterial communities found in the ectomycorrhizosphere of *Xerocomus pruinatus* and *Scleroderma citrinum* on oak were similar at the genus level, but different at the OTU level, demonstrating the specificity of the ectomycorrhizosphere. In the future, further refinements to molecular techniques, enhanced bioinformatic analysis and development of novel methods to culture and study these newly revealed organisms should enable links between these organisms and their functions to be elucidated. To date most of our knowledge on the role of associated microorganisms in the ectomycorrhizosphere has been based on studies of culturable organisms. The spectrum of plant-microbe relationships in the rhizosphere can range from mutualistic to pathogenic (Bais et al., 2006). Plant-growth-promoting rhizobacteria (PGPR)—which are found in the rhizosphere and mycorrhizosphere—benefit plants by creating biofilms that protect the root against pathogens (Akhtar and Siddiqui, 2009). These rhizosphere bacteria induce systemic acquired resistance (preparing the plant for attack; Pieterse et al., 2003) and enhance plant growth (Adesemoye et al., 2008; Yang et al., 2009). Several very good reviews have been written on PGPR (Vessey, 2003; Lugtenberg and Kamilova, 2009).There is some evidence of synergistic interactions between PGPR and mycorrhizal fungi, which may benefit the plants as a result of greater nutrient acquisition, inhibition of plant pathogens and greater mycorrhization (Artursson et al., 2006). Uroz et al. (2007) demonstrated positive interactions between ECM and bacteria that result in increased weathering of mineral nutrients, ultimately increasing nutrient uptake by the plant. AM have also been found to alter the structure of mycorrhizosphere microbial communities (Rillig and Mummey, 2006; Toljander et al., 2007; Welc et al., 2012). Isolation and identification of rhizobacteria found in the mycorrhizosphere around AM hyphae have shown bacteria with antagonistic properties toward soil-borne pathogens (Lioussanne et al., 2010), and antifungal properties (although they do not affect the AM symbiosis; Dwivedi et al., 2009). The N_2_ fixing ability of some AM plants improves when mycorrhizae are present vs. when they are absent (Kucey and Paul, 1982; Fitter and Garbaye, 1994).

Greater mycorrhization effects have been attributed to one specific group of PGPR, the so-called mycorrhization-helper bacteria (MHB) (Garbaye, 1994). Three life-stages in mycorrhizal fungi have been recognized, the free-living saprotrophic, the pre-infection stage and the symbiotic, mycorrhization stage (Deveau et al., 2007; Courty et al., 2008). During the pre-infection “free-living stage,” mycorrhizal fungi can interact with specific bacteria (e.g., *Pseudomonas* species) that are thought to enhance mycorrhizal establishment (Garbaye, 1994; Pivato et al., 2009). These mycorrhization-helper bacteria (MHB) can increase mycorrhization of a plant 1.2–17.5 times (Frey-Klett et al., 2007). Mycorrhization-helper bacteria are not plant-specific, but may be fungal-specific (Garbaye, 1994; Pivato et al., 2009). For example, *Pseudomonas fluorescens* BBc6R8 promotes survival of ECM *Laccaria bicolour* S238N when in its free-living stage, increasing radial fungal growth, hyphal density and branching angle. Mycorrhization-helper bacteria also change mycelial physiology from the free-living saprotrophic state to a “pre-symbiotic” stage (Deveau et al., 2007). During mycorrhization, a proliferation of bacteria can improve the receptivity of roots (Aspray et al., 2006), accelerate germination of fungal propagules in soil (Garbaye, 1994), and increase production of compounds such as auxofurans (Tylka et al., 1991) which have been shown to affect fungal metabolism and gene expression (Riedlinger et al., 2006). Mycorrhization-helper bacterial strains identified thus far include: Gram-negative Proteobacteria, Gram-positive Firmicutes and Gram-positive Actinomycetes (Frey-Klett et al., 2007) (Table 3). How MHB encourage mycorrhization is only beginning to be unraveled. Most MHB increase fungal colonization of the roots via: stimulating mycelia extension and branching (Garbaye, 1994; Poole et al., 2001; Schrey et al., 2005), increasing root-fungus contacts/colonization, and influencing soil environmental conditions (Frey-Klett et al., 2007). Mycorrhization-helper bacteria have been observed to stimulate spore germination of *Glomus mosseae* and *Glomus clarum* (AM) (Mosse, 1962; Xavier and Germida, 2003, respectively). In the case of *Glomus clarum* there may have been a complex bacterial consortium producing antagonistic volatiles (Tylka et al., 1991). The release of a number of different compounds, including gasses (Duponnois and Kisa, 2006) and secondary metabolites (e.g., auxofuran)(Keller et al., 2006; Riedlinger et al., 2006) by MHB have been shown to increase mycelial growth. Mycorrhization-helper bacteria are thought to reduce plant and mycorrhizal stress by detoxifying soil (e.g., Polyphenolic substances produced by *Paxillus involutus* are toxic to the fungus, but can be broken down by MHB; Duponnois and Garbaye, 1990). The potential for MHB to increase and support mycorrhizal infection has been demonstrated only under laboratory conditions. However, as in the case of PGPR, little is known about the effect these bacteria have on mycorrhization *in situ*.

### Tree-mycorrhizal-microbial and faunal interactions

The term rhizosphere fauna has typically been used to refer to agricultural pests, specifically root herbivores (Bonkowski et al., 2009). However, rhizosphere fauna encompass a broad range of feeding types, including those that feed on bacteria, mycelium, and other fauna. Soil fauna influence the composition and activity of microbial populations by: directly grazing on bacteria and fungal hyphae, transporting fungal and microbial cells in their gut (thus facilitating microbial dispersion) and changing physical and chemical conditions of the soil (i.e., worm casts) (Oades, 2003). Several fungivorous collembola species have the capacity to influence development of Basidiomycete mycelia (Tordoff et al., 2008; Crowther et al., 2011b), and the extent of that influence is directly dependent on collembola density (Hanlon and Anderson, 1979; Kaneko et al., 1998). Setälä (1995) compared control soil (no fauna) and faunal-inoculated soil in Scots pine and silver birch microcosms. In all cases the presence of soil fauna reduced ECM abundance, reduced microbial biomass and increased shoot production. Faunal community impacts on decomposer fungi have also been shown to be density dependent, although there is evidence that the faunal community composition may have a greater impact on the microbial community. Crowther and A'Bear (2012) found that grazing pressures exerted by low-density woodlouse populations on saprotrophic fungi surpassed grazing pressures exerted by high density millipedes or high density collembola populations, ultimately limiting mycelial development. Grazing of mycelium not only influences microbial populations, but also has direct impacts on nutrient cycling because it increases enzyme release into the mycorrhizosphere (Crowther et al., 2011a), particularly in the presence of macrofauna (Crowther et al., 2011b). This increase will, in turn, affect soil nutrient availability (both N and P) and SOM turnover.

Mycophagous soil fauna grazing on AM and ECM in forests will affect C flow into the mycorrhizosphere by disrupting the movement of C along rhizomorphs and runner-type hyphae (Setälä, 1995; Coleman et al., 2004). Once removed from its C source, the growing hyphal front will stop releasing exudates, stop growing and potentially die off or convert to a saprotrophic life stage. The amount of hyphal grazing varies with mycorrhizal species, as soil fauna have been shown to be selective in their feeding preferences (Klironomos and Kendrick, 1996; Crowther and A'Bear, 2012). Klironomos and Kendrick (1996) showed that mites and collembola preferentially graze fungi growing on litter. However, when offered only AM growing on maple, they consume the fine hyphae most distant from the root. Cesarz et al. (2013) demonstrated that ECM vs. AM mycorrhizal-tree identity had a major influence on belowground nematode communities. Ash, which forms AM symbiosis, had greater populations of bacterial-feeding nematodes and lesser populations of fungal-feeding nematodes. In contrast beech, which forms EM symbiosis, had enhanced fungal-feeding nematode populations. Grazing on hyphal mycorrhizal networks can also significantly influence plant-C allocation belowground, and may influence C sequestration (Johnson et al., 2005).

## Consequences of the mycorrhizosphere on soil C

Differences in tree-mycorrhizal symbiosis types may impact C-cycling and C sequestration because of differences in C allocation and longevity of these structures in soil. ECM trees with extensive mycelia have two to three times more C flux to the soil than AM trees (Finlay and Söderström, 1992; Phillips and Fahey, 2005; Pumpanen et al., 2009). This may be due to ECM roots being “more leaky,” possibly due to higher exudation rates (Phillips and Fahey, 2006). Pumpanen et al. (2009) demonstrated that roots and ECM growth account for 13–21% of recently assimilated C, whereas 9–26% of recently assimilated C is respired from the roots and rhizosphere. The turnover times of ECM and AM are also dramatically different; the turnover times of EMM and mycorrhizal fine roots are in the order of months to years (Cairney, 2012) and AM days to weeks (Langley and Hungate, 2003). The slower turnover of ECM is thought to be due to the chitin content of this fungal tissue, although in AM fungi the production of the glycoprotein glomalin can decrease AM hyphal turn over times significantly. Glomalin binds the soil matrix forming a soil aggregate within which AM hyphae are trapped and are slow to decompose, having an estimated residence time of 6–42 years (Rillig, 2004). These soil aggregates represent more the 5% of total soil C, significantly contributing to long term soil C sequestration (Wright and Upadhyaya, 1998; Rillig et al., 2001). However, the grazing of AM fungi is also higher than ECM because of the thin walls of AM fungi, which reduces residence times of this C in soil (Klironomos and Kendrick, 1996). Cheng et al. (2012) recently suggested that AM fungi diminish rather than enhance soil C pools in the short-term, as a result of accelerated decomposition of litter, when sites are exposed to elevated CO_2_. Although ECM production of proteolytic and lignolytic enzymes enables increased degradation of SOM (releasing more C) relative to AM (Read, 1992; Chalot and Brun, 1998), the recent study by Clemmensen et al. (2013) has suggested that accumulation and preservation of root and root-associated fungal residues is responsible for up to two-thirds of the C sequestered in boreal forests. This suggests that ECM dominated soils are more likely to sequester soil C, at least in the short term. However, long-term effects (decadal) may be qualitatively different from short-term effects; specifically there may be a long-term gain in recalcitrant compounds (Verbruggen et al., 2012).

ECM differ in nutrient uptake and transfer rates, altering the net primary production (NPP) of trees and may ultimately influence ecosystem C-cycling and C sequestration (Burgess et al., 1993). The ability of ECM to promote tree NPP varies depending on the extent of root colonization, the type of hyphae (Colpaert et al., 1992; Thomson et al., 1994) and the ability of the hyphae to acquire and transfer nutrients to the tree (Agerer, 2001). ECM fungi also have broad enzymatic capabilities (Chalot and Brun, 1998) that allow them to decompose labile and recalcitrant components of SOM, access organic sources of N, and transfer large amounts of N to host plants (Hobbie and Hobbie, 2006). AM fungi can also acquire substantial N from SOM (Hodge et al., 2010; Whiteside et al., 2012), although they do not have as broad an N-based enzymatic capability and appear to transfer only a small fraction of their host plants demand for N (Hodge and Fitter, 2010). This is particularly evident in dry soil conditions when N transport by roots is restricted, but soil N levels are still high (Tobar et al., 1994; Govindarajulu et al., 2005). There is some evidence that AM hyphae hydrolyze organic C at their root tips (Koide and Kabir, 2000), but there is limited evidence of AM derived phosphatases in the mycorrhizosphere along the hyphae (Joner et al., 2000). Belowground C allocation in AM-fungal-dominated ecosystems may not return sufficient N (or P) to offset the C investment by the tree, limiting the increase in NPP associated with greater atmospheric CO_2_ concentrations (Drake et al., 2011). Turnover of SOM has been shown to be faster in forest stands with AM mycorrhizal associations compared to ECM (Vesterdal et al., 2012). Phillips et al. (2013) proposed that forests dominated by AM and ECM associated trees vary in their C cycling and nutrient acquisition and may respond to global changes in predictable ways. They have proposed a new framework for predicting these variations in biogeochemical processes between forests [the Mycorrhizal-Associated Nutrient Economy model (MANE)] using forest inventory analysis maintained by the US Forest Service and previously described mycorrhizal designations (Brundrett et al., 1990; Wang and Qui, 2006). AM-dominated forest stands will have an inorganic nutrient economy resulting from elevated rates of C, N, and P mineralization and high quality litter. In contrast, ECM-dominated forest stands will have an organic nutrient economy as a result of slow rates of C, N, and P turnover and a lower quality litter. Thus further supports the hypothesis that ECM dominant forests will sequester more C.

ECM have the potential to act as a strong C sink, acquiring large amounts of C from their plant hosts (Smith and Read, 2008). The ECM then move the plant C to their hyphal tips, generating new biomass and exuding various compounds for nutrient acquisition. This movement of C can be a significant transport of plant C beyond the rhizosphere (Norton et al., 1990; Erland et al., 1991; Finlay and Söderström, 1992), and the recalcitrant chitinous cell wall of the mycelium will remain in the soil for months (Setälä et al., 1999; Treseder and Allen, 2000). The life-span of ECM root tips may be anywhere from 3 to 22 months (Orlov, 1960; Majdi et al., 2001), and may increase with soil depth (Pritchard et al., 2008a; McCormack et al., 2010). The consequences of C movement throughout the soil via hyphae is only beginning to be understood. Carbon will be transported out of the rhizosphere, moving as little as a few centimeters to as much as tens of meters (Gryta et al., 1997; Dunham et al., 2003; Murata et al., 2005; Churchland et al., 2012). However, long-distance, continuous, transport of C in hyphae is likely small as EMM are often fragmented, due to foraging by soil fauna (Dahlberg and Stenlid, 1995) and there are impermeable cell walls that form physiologically separated regions along hyphae (Olsson, 1999). However, C movement along hyphae would be very difficult to measure, and the potential impacts of the movement on C sequestration is large. Depending on forest type, climate and measurement methods, estimates of fungal biomass in ECM root tips can range from 20–10,000 kg/ha (Fogel and Hunt, 1979; Vogt et al., 1982; Dahlberg et al., 1997; Satomura et al., 2003; Sims et al., 2007; Helmisaari et al., 2009; Okada et al., 2011). The majority of this biomass is found in the forest floor and organic soil layers (Bååth et al., 2004; Wallander et al., 2004; Göransson et al., 2006), and constitute up to 1/3 of the total microbial biomass in forests (Swedish conifer forest; Högberg and Högberg, 2002). A recent study by Clemmensen et al. (2013) determined that 50–70% of stored C belowground was derived from root and root-associated microorganisms. Using ^14^C bomb-carbon modeling Clemmensen et al. (2013) found preservation of fungal residues in late-successional forests and in particular root-associated fungi, not saprotrophs, are the important regulators of ecosystem C dynamics. The sheer volume of tree C allocated belowground, and the ability of this C to move throughout the soil profile and soil ecosystem, shows how important it is to determine accurate C models of forests and other mycorrhizal-dominated soil ecosystems.

## Conclusions

Carbon allocation to mycorrhizal hyphae enhances the degree to which tree C can impact soil microbial communities and soil C cycling. Different mycorrhizal morphotypes will vary the spatial distribution of this C considerably, although the vast, delicate nature of mycorrhizal hyphae makes this a difficult area of study. The greater C allocation to mycorrhizal roots, coupled with slower turnover times of mycorrhizal roots compared to non-mycorrhizal roots, hints at the potential of mycorrhizal associations to increase C sequestration in soil. However, differences between ECM and AM may impact soil C sequestration. ECM roots have longer turnover times than AM, and, due to their chitinous cell walls, are less likely to be grazed by fauna. Recent improvements in stable-isotope labeling and probing methods have resulted in a better understanding of the quantity and quality of C exuded belowground and spatial and temporal dynamics of C flow in forest soil. In addition whole-genome sequencing is showing us the large suite of important genes and signals involved in symbiotic associations. These new techniques should enable great strides to be made on our understanding of the role of different mycorrhizal functional groups in forest C cycling. We suggest the next step in developing this understanding would be tracing the flow C throughout the different hyphal morphotypes and measuring turnover times of this C to establish how ECM and AM distribute C in forest soil. This could be done through a combination of stable-isotope labeling and probing techniques conducted in large-scale controlled conditions, such as a biotron, coupled with nanosims technology to increase sensitivity and isotopic detection at high spatial resolution. We may then be able to determine the consequences of these variations for C-cycling and C sequestration.

### Conflict of interest statement

The authors declare that the research was conducted in the absence of any commercial or financial relationships that could be construed as a potential conflict of interest.
